# Structural Features of the [C4mim][Cl] Ionic Liquid and Its Mixtures with Water: Insight from a ^1^H NMR Experimental and QM/MD Study

**DOI:** 10.1021/acs.jpcb.1c08215

**Published:** 2021-11-22

**Authors:** Dovilė Lengvinaitė, Sonata Kvedaraviciute, Stasė Bielskutė, Vytautas Klimavicius, Vytautas Balevicius, Francesca Mocci, Aatto Laaksonen, Kęstutis Aidas

**Affiliations:** † Institute of Chemical Physics, Faculty of Physics, Vilnius University, Vilnius LT-10257, Lithuania; ‡ DTU Chemistry, Technical University of Denmark, Kongens Lyngby DK-2800, Denmark; § Università di Cagliari, Dipartimento di Scienze Chimiche e Geologiche, Cittadella Universitaria di Monserrato, Cagliari I-09042, Monserrato, Italy; | Energy Engineering, Division of Energy Science, Luleå University of Technology, Luleå 97181, Sweden; ⊥ Division of Physical Chemistry, Department of Materials and Environmental Chemistry, Arrhenius Laboratory, Stockholm University, Stockholm 10691, Sweden; # Center of Advanced Research in Bionanoconjugates and Biopolymers, “Petru Poni” Institute of Macromolecular Chemistry, Iasi 700469, Romania; ¶ State Key Laboratory of Materials-Oriented and Chemical Engineering, Nanjing Tech University, Nanjing 211816, China

## Abstract

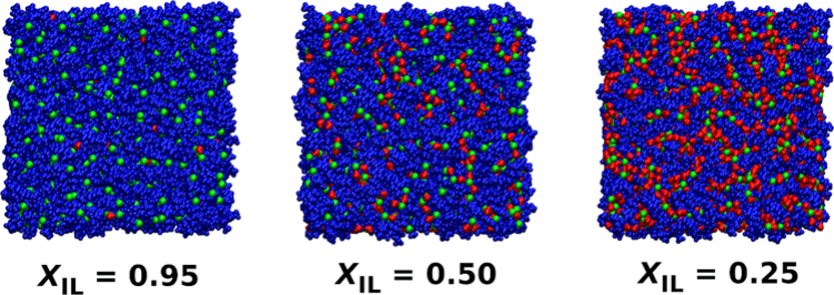

The ^1^H NMR chemical shift of water exhibits non-monotonic dependence on the composition of an aqueous mixture of 1-butyl-3-methylimidazolium chloride, [C4mim][Cl], ionic liquid (IL). A clear minimum is observed for the ^1^H NMR chemical shift at a molar fraction of the IL of 0.34. To scrutinize the molecular mechanism behind this phenomenon, extensive classical molecular dynamics simulations of [C4mim][Cl] IL and its mixtures with water were carried out. A combined quantum mechanics/molecular mechanics approach based on the density functional theory was applied to predict the NMR chemical shifts. The proliferation of strongly hydrogen-bonded complexes between chloride anions and water molecules is found to be the reason behind the increasing ^1^H NMR chemical shift of water when its molar fraction in the mixture is low and decreasing. The model shows that the chemical shift of water molecules that are trapped in the IL matrix without direct hydrogen bonding to the anions is considerably smaller than the ^1^H NMR chemical shift predicted for the neat water. The structural features of neat IL and its mixtures with water have also been analyzed in relation to their NMR properties. The ^1^H NMR spectrum of neat [C4mim][Cl] was predicted and found to be in very reasonable agreement with the experimental data. Finally, the experimentally observed strong dependence of the chemical shift of the proton at position 2 in the imidazolium ring on the composition of the mixture was rationalized.

## Introduction

While earlier often regarded as an undesirable contaminant,^1,2^ water has over the last decade been increasingly considered as an integral part of ionic liquid (IL) or more broadly salt-based materials, greatly extending their limits of functionalization and applicability.^3,4^ The mixtures of IL and water may acquire unique properties which are not necessarily associated with any of the two components.^5−9^ For example, hydrated choline dihydrogenphosphate was found to be a superior solvating medium for some proteins as compared to the ordinary aqueous buffer solutions,^5,6^ not only suppressing protein denaturation over significantly extended periods of time but also preserving their function.^7^ The mixtures of phosphonium IL and water can be used for an effective extraction of water-soluble proteins through a temperature-controlled reversible transition between homogeneous and separated liquid–liquid phases.^3,10^ Hydrated eutectic melts of metallo-organic salts have demonstrated the potential to be used as stable electrolytes in high-energy-density halide-free aqueous batteries.^4,11,12^

To disclose the molecular mechanisms behind the physicochemical properties of IL/water mixtures, various experimental and theoretical techniques have been called in order to provide a detailed insight into the molecular structure and dynamics of these heterogeneous systems.^8,13−15^ In particular, classical molecular dynamics (MD) simulations have led to the general conclusion that solitary water molecules are dispersed throughout the bulk of the IL when water content is rather low.^14,16−21^ Under these circumstances, the isolated water molecules are found to primarily form hydrogen bonds with the anions,^16,17,21^ acting as bridges between them.^19,20,22^ When the molar fraction of water increases, clusters of water molecules begin to emerge,^17,18,22^ and nanostructural organization of the mixture is enhanced.^19,23^ Eventually, a continuous water network is formed, which percolates the entire system and surrounds the ionic clusters.^18,20−22^

Water is thus often seen as providing a medium that screens electrostatic interactions between ions, with an immediate implication of reduced viscosity of the liquid—an often desired property that facilitates practical applications. However, a possibility for a rather peculiar agglomeration of water molecules into long-lived water pockets confined within the IL matrix was hinted by X-ray and neutron scattering as well as by nuclear magnetic resonance (NMR) experiments.^24,25^ It is claimed that these water pockets form when molar fraction of water, χ_w_, is in the range of 0.70–0.90,^26^ and their averaged size was estimated to be around 20 Å.^27^ Although the concept of the water pocket has recently received some criticism,^28^ polarizable MD simulations do indicate such a possibility, albeit in the case of hydrophobic anionic constituents of the IL.^29^

**Figure 1:**
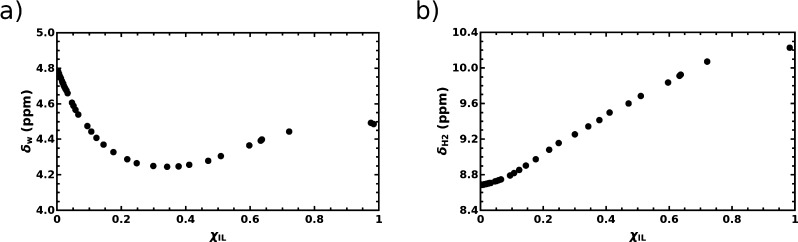
^1^H NMR chemical shift of water, δ_w_, (a) and of the proton at position 2 in the imidazolium ring of the C4mim^+^ cation, δ_H2_, (b) as a function of the molar fraction of the IL, χ_IL_, in the mixture of [C4mim][Cl] IL and water.

In this work, precise measurements of water ^1^H NMR chemical shifts for the aqueous mixtures of 1-butyl-3-methylimidazolium chloride, [C4mim][Cl], were performed varying the molar fraction of the IL, χ_IL_, in the range of 10^–3^ to 0.98. At the lowest molar fraction of the IL, water dominates the sample, and thus, a measured chemical shift of water of 4.8 ppm is virtually the same as that of neat water at ambient conditions.^30,31^ As illustrated in Figure 1a, a clear non-monotonic evolution of the ^1^H NMR chemical shift of water with the increasing molar fraction of the IL was recorded in this work.

Our experimental findings thus indicate that the assumption of a converging ^1^H NMR chemical shift of water that was thought to be a manifestation of the formed water pockets in the aqueous mixtures of [C4mim][Cl] in ref (32) is in fact inappropriate. The data in Figure 1a suggests that the chemical equilibrium between various water-ionic and water–water molecular aggregates may vary with the composition of the mixture in a more delicate manner than assumed previously. Interestingly, a smooth increase of the ^1^H NMR chemical shift of the proton at position 2 in the imidazolium ring, H_2_, was observed previously for the increasing molar fraction of the IL in the [C4mim][Cl]/water mixture^33^ and that is also confirmed by our measurements as shown in Figure 1b. The shape of the curve in Figure 1b could simply imply that water molecules are gradually replacing chloride anions in the vicinity of the C_2_–H_2_ moiety with the increasing content of water in the mixture,^34^ thus potentially hiding the possible occurrence of more intricate structural reorganizations. Indeed, the increasing chemical shift of water seen in Figure 1a for χ_IL_ in the range of 0.34–0.98 can be expected due to the relative proliferation of water molecules bound to strongly hydrophilic chloride anions. The decrease of the chemical shift of water in the mixture as compared to that of neat water observed for χ_IL_ in the range 0–0.34 in Figure 1a is, however, rather perplexing. To the best of our knowledge, similar dependence as in Figure 1a was first observed for the mixture of 1-butyl-3-methylimidazolium nitrate and water by Bystrov et al.,^28^ yet it was not analyzed in depth.

To provide rationalizations of experimental NMR data of IL systems, computational strategies of different levels of sophistication have been previously applied.^35−41^ Notably, a viable route to achieve accurate predictions of the ^1^H NMR spectrum of imidazolium IL as well as to rationalize its shape was demonstrated in refs (42) and (43), where classical or quantum MD simulations were performed, and condensed-phase chemical shifts were obtained as time averages over molecular trajectory. Very recently, Saielli combined classical MD simulations with quantum ONIOM calculations of NMR chemical shifts of the [C4mim][Cl] IL and its mixtures with water.^44^ Even though the accuracy of predicted NMR spectra based on classical trajectories was questioned previously,^43^ an important insight into the molecular structure of the studied IL systems was reached.^44^ We have employed classical MD simulations along with a combined quantum mechanics/molecular mechanics (QM/MM) model to study various NMR parameters^45−48^ and lately addressing ion pairing in the solutions of [C10mim][Cl] IL where solvents differ in polarity and capabilities for hydrogen bonding.^49^ In this work, this computationally expensive yet accurate computational technique is applied in order to gain molecular-level insight into structural organization of [C4mim][Cl]/water mixtures reflecting experimental NMR data in Figure 1.

## Methods

### MD Simulations

All MD simulation runs and subsequent structural analyses have been carried out using the Amber18 suite of programs.^50^ A Canongia-Lopes et al. potential was applied for the constituent ions of the [C4mim][Cl] IL,^51−53^ and the TIP4P-Ew force field was selected for water molecules.^54^ Neat [C4mim][Cl] IL was simulated as a system of 1000 [C4mim][Cl] ion pairs. An appropriate number of water molecules of 48, 1000, or 3000 were added to the 1000 ion pairs to get mixtures with a molar fraction of the IL of 0.95, 0.50, or 0.25, respectively. The system of pure water was represented by 1000 TIP4P-Ew water molecules. The initial configuration of each simulated system was generated by placing ions and water molecules randomly in the simulation box by using the Packmol program.^55^

For the systems of neat IL and its mixtures with water, a short simulation of 50 ps in the *NVT* ensemble was executed at a temperature of 50 K after initial energy minimization run. A series of 10 500 ps-long simulations in the *NPT* ensemble followed where the temperature was increased incrementally in steps of 50 K from 50 to 500 K. At 500 K, systems were simulated for 4 ns in the *NPT* ensemble. After that, all systems were cooled down to 298 K in steps of 50 K where the duration of each *NPT* simulation was 500 ps again. At ambient conditions, each system was simulated for 17 to 20 ns in the *NPT* ensemble to get converged mass densities. Then, the simulations in the *NVT* ensemble followed for 12 ns, and the trajectories recorded during the last 5 ns of the simulation were used in the structural analyses as well as for the QM/MM calculations. A similar protocol was executed to simulate pure water, just all simulations in this case have been carried out at ambient conditions only, and the equilibration in the *NPT* ensemble for 500 ps was sufficient. A final simulation of water at ambient conditions was carried out for 2.5 ns in the *NVT* ensemble.

MD simulations were carried out using the Sander module of Amber18. Periodic boundary conditions were employed, and a cut-off of 12 Å was used for nonbonded interactions. The SHAKE algorithm^56^ was imposed to constrain all bonds involving hydrogen atoms. The equations of motion were integrated using the leap-frog algorithm with a time step of 1 fs. The temperature was controlled using the Langevin thermostat with a collision frequency of 3.0 ps^–1^. The pressure was set to 1 bar in all MD simulations. Configurations were dumped at regular intervals of 10 ps during the production run phase.

### QM/MM Calculations

The QM/MM method^57,58^ based on the gauge including the atomic orbital approach and density functional theory as implemented in the Dalton electronic structure program^59^ has been used for the calculations of NMR isotropic shielding constants in this work. The PBE0 exchange–correlation functional^60^ and the Ahlrichs def2-TZVP basis set^61^—occasionally combined with the Pople style 3-21G basis^62^—were applied for the QM subsystem of the model. The classical subsystem was represented by point charges. The point charges for the C4mim^+^ cations were derived by using the restrained electrostatic potential procedure, RESP,^63^ implemented in the Antechamber module^64^ of Amber18. The Hartree–Fock approach along with the 6-31G* basis set was used to optimize the geometry of the C4mim^+^ cation and to compute potentials to be utilized in the RESP procedure using Gaussian 09 programs.^65^ Point charges from the TIP3P potential^66^ were used for water molecules. Condensed-phase results for NMR shielding constants are obtained as statistical averages over 100 molecular configurations selected from the MD trajectories at regular intervals of 50 ps for IL systems and of 20 ps for liquid water. We have applied a spherical cut-off radius centered at the center of mass of the central cation or water molecule for every molecular configuration, and the cut-off radius was set to 30 and 15 Å for IL systems and liquid water, respectively. The QM region was expanded to include some of the ions or water molecules around the central species as indicated for each specific system below.

## Experimental Section

All NMR measurements were performed on a Bruker AVANCE III HD 400 MHz NMR spectrometer using a Bruker Ascend 9.4 T superconducting magnet and a 5 mm broad band outer probe. For ^1^H NMR measurements, 16 scans were accumulated, the temperature was stabilized at 25 ± 0.1 °C, and the repetition delay was set to 5 s. A capillary insert filled with D_2_O and sodium trimethylsilylpropanesulfonate (DSS) mixture was used for magnetic field stabilization and chemical shift referencing. IL [C4mim][Cl] was obtained from Sigma-Aldrich (99% purity) and was used without further purification. Aqueous IL mixtures were prepared using deionized water directly in NMR tubes by weighting the components using KERN ABJ-NM/ABS-N analytical balances (±0.1 mg). In the case of spectral overlapping to obtain correct chemical shift values, spectral fitting using OriginPro 9 software was performed.

## Results and Discussion

### Neat [C4mim][Cl] IL

#### Structural Analysis

**Figure 2:**
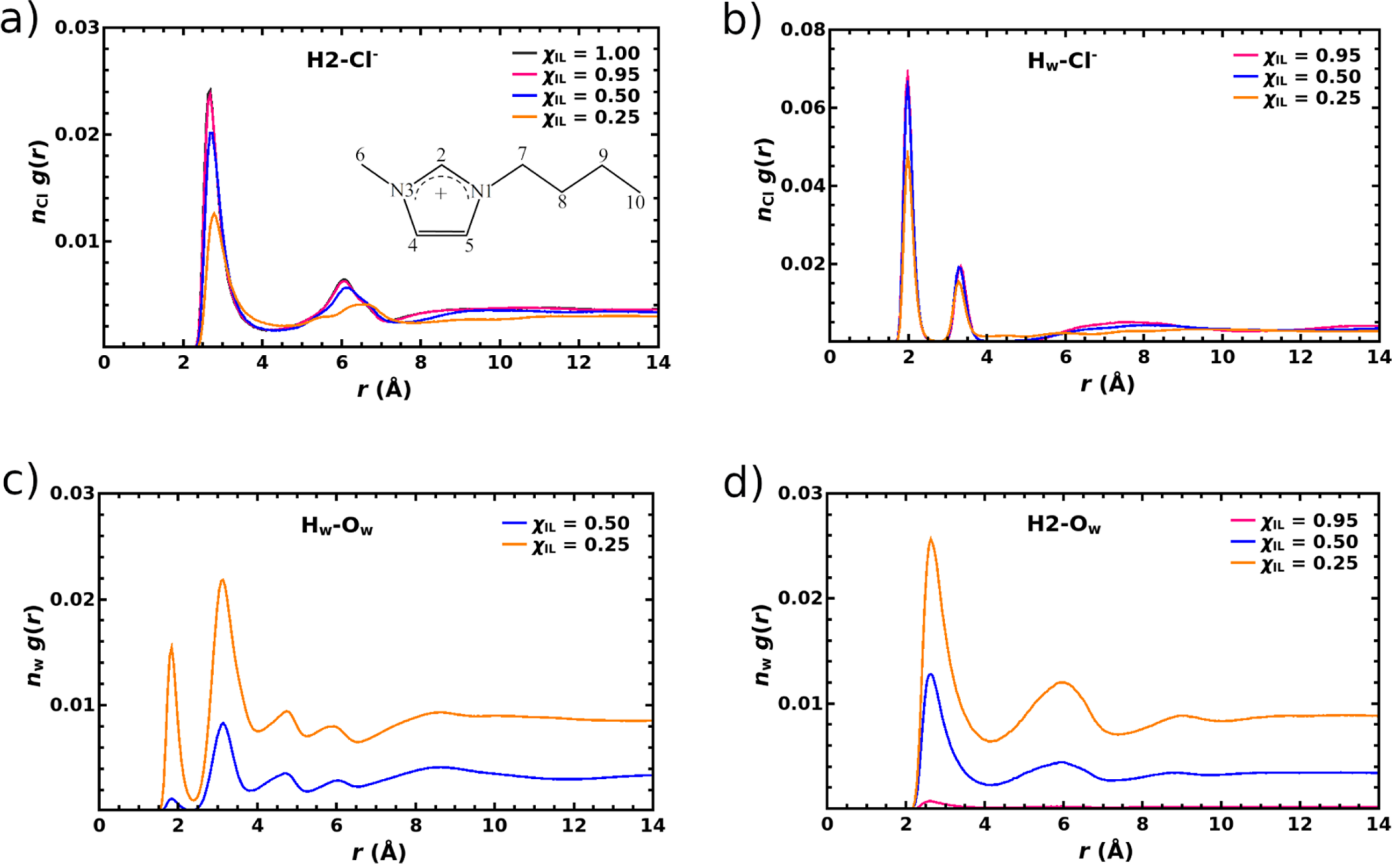
(a) RDFs between the H_2_ atom and the Cl^–^ ion in neat [C4mim][Cl] IL and its mixtures with water, all scaled by the number density of the chloride anions, *n*_Cl_. Atom numbering in the C4mim^+^ cation is included as well. (b) RDFs between hydrogen atoms of water and Cl^–^ ions in neat [C4mim][Cl] IL and its mixtures with water, all scaled by *n*_Cl_. (c) RDFs between hydrogen and oxygen atoms of water in the mixtures of [C4mim][Cl] and water, all scaled by the number density of water, *n*_w_. (d) RDFs between the H_2_ atom of C4mim^+^ cation and oxygen atoms of water, all scaled by *n*_w_.

First focusing on the structural features and ^1^H NMR spectrum of neat [C4mim][Cl] IL, we show in Figure 2a the radial distribution function (RDF) between the H_2_ hydrogen atom in the C4mim^+^ cations and the chloride anions. Atom numbering in the C4mim^+^ cation is also presented in Figure 2a. The RDFs in this work are scaled by appropriate number densities to facilitate the comparison of interionic and intermolecular distributions across systems of different compositions, as proposed in ref (29). A pronounced peak between 2.2 and 4.0 Å in Figure 2a demonstrates a prolific hydrogen bonding interaction between the C_2_–H_2_ moiety of imidazolium and the chloride anions,^67^ and the most probable H_2_··· Cl^–^ distance is found to be 2.67 Å. The spherical integration of that peak up to 4 Å gives a coordination number of 1.47. Chlorides are also found to coordinate imidazolium in the vicinity of the C_4_–H_4_ and C_5_–H_5_ bonds. We refer to the Supporting Information for the structural analysis of distribution of ions and water molecules around the C_4_–H_4_ and C_5_–H_5_ moieties of the C4mim^+^ cations. Our MD results confirm evidently the greater capacity of the C_2_–H_2_ moiety for hydrogen bonding as compared to that of C_4_–H_4_ or C_5_–H_5_.^36,67^

**Figure 3:**
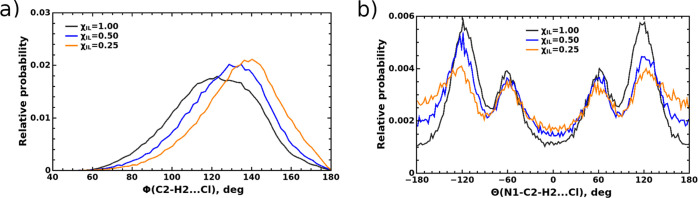
Distributions of (a) C_2_–H_2_··· Cl^–^ angle, Φ, and (b) N_1_–C_2_–H_2_··· Cl^–^ dihedral angle, Θ, in neat [C4mim][Cl] IL and its mixtures with water for *R*(H_2_···Cl^–^)≤4.0 Å.

To scrutinize the structural distribution of anions around the imidazolium ring in more detail, we show in Figure 3a the distribution of angles between the C_2_–H_2_ bond in the imidazolium ring and the Cl^–^ anions that are found within the sphere of 4 Å radius centered at the H_2_ atom.

**Figure 4:**
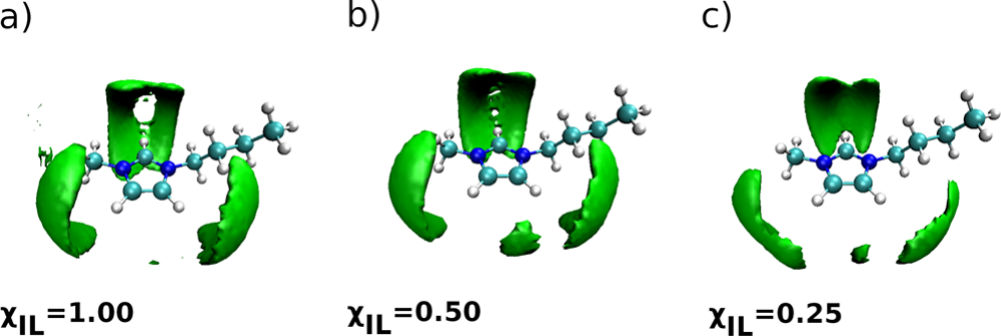
SDF of Cl^–^ anions around the imidazolium ring of the C4mim^+^ cation in (a) neat [C4mim][Cl] IL and its mixtures with water at χ_IL_ of (b) 0.50 and (c) 0.25.

It is evident that the hydrogen bond formed between the C_2_–H_2_ moiety and chloride is strongly non-linear, and thus, the configuration of the linear C_2_–H_2_··· Cl^–^ hydrogen bond, which corresponds the global minimum of the potential energy surface for the isolated C4mim^+^–Cl^–^ ion pair,^68^ is virtually absent in the neat liquid. Some of the chloride anions are seen to approach the imidazolium ring from the top of the C_2_–H_2_ bond, and yet the most probable C_2_–H_2_··· Cl^–^ angle lies in the range of around 110–140 deg. These findings are in line with the results of neutron diffraction measurements, which indicate the non-linear hydrogen bond between the C_2_–H_2_ moiety and chloride in the samples of 1,3-dimethylimidazolium chloride^69^ and with the results from Car-Parinello MD simulations that showed the most prominent maximum in the same interval.^70^ The distribution of the N_1_–C_2_–H_2_··· Cl^–^ dihedral angle recorded in neat [C4mim][Cl] clearly shows that chloride anions tend to be located out of the plane of the imidazolium ring, see Figure 3b. The distribution is symmetric with respect to the center point at 0 deg, and pronounced peaks at ± 120 deg indicate that anions rather prefer to stay on the methyl side of the C_2_–H_2_ bond. The location on the butyl side is less preferred as indicated by smaller peaks at ±60.0 deg, likely due to the steric hindrance. These findings are confirmed evidently by the spatial distribution function (SDF) of chloride anions around the imidazolium ring of the C4mim^+^ cation shown in Figure 4a.

#### NMR Results

The structural features of neat [C4mim][Cl] IL discussed above will definitely shape its ^1^H NMR spectrum because the calculated NMR chemical shift, in particular, of H_2_ can differ by as much as 8–9 ppm between in-plane and on-top of the C_2_–H_2_ bond complexes of the isolated geometry-optimized ion pair of imidazolium cation and chloride anion.^37,43^ In addition, it has been suggested that the ^1^H NMR spectrum of the imidazolium cation is mainly determined by the ions and molecules in the immediate vicinity of the cation, and the effect of ions beyond the first solvation shell is very small.^39,44^ Our QM/MM results for the ^1^H NMR isotropic shielding constants in neat [C4mim][Cl] IL are collected in Table S1 of the Supporting Information. According to the most basic computational scheme, only the central cation is treated at the density functional theory (DFT) level in every configuration while all other ions are represented by the point charge potential. To evaluate the direct effect of the environment on the shielding constants of the C4mim^+^ cation, we have also performed calculations on the isolated central cation in the same set of configurations, that is, with all other ions removed. As can be seen in Table S1, the direct effect of the environment represented by point charges is to reduce the shielding constants of protons in the imidazolium ring by 0.3–0.4 ppm, while the effect on the shieldings of hydrogen atoms in the methyl and butyl groups is smaller and practically does not exceed 0.2 ppm. Because the non-electrostatic effects on the NMR properties of molecules involved in hydrogen bonding are typically important,^48,58,71^ we have also performed QM/MM calculations where three additional ions were promoted to the QM region of the model, those which are closest to the H_2_, H_4_, and H_5_ atoms of imidazolium. We observed in Table S1 that this improvement of the model leads to further reduction of the shielding constants of H_2_, H_4_, and H_5_ atoms by as much as 0.4–0.5 ppm. To obtain a converged effect, further expansion of the QM region around the central cation is necessary, but this also implies a much increased computational burden. However, it was very recently reported that the satisfactory results for the ^1^H NMR shielding constants of [C4mim][Cl] IL can be obtained if the modest 3-21G basis set is used for the ions around the central cation.^44^ We thus have repeated the QM/MM calculations by keeping the def2-TZVP basis set for the central cation and switching to the 3-21G basis for the same three ions around the acidic hydrogens of the imidazolium ring as in previous calculations. Despite the reduced flexibility in the description of the electronic density of the additional ions, the results are virtually identical not only for the statistically averaged shieldings as can be seen in Table S1 but rather astonishingly also for the shielding constants in all individual configurations. However, this effect is clearly due to the fortuitous cancellation of errors as improving the 3-21G basis set by the diffuse functions leads to larger discrepancies for shieldings of atoms H_2_, H_4_, and H_5_ as compared to the case where def2-TZVP basis was used for all ions in the QM region of the model, see Table S1. These findings in a way echo former observations that quantum chemical predictions of NMR chemical shifts using small basis set can in some cases lead to much better agreement with experimental data than those that employ extensive basis sets.^72,73^

Using the 3-21G basis set for all additional ions around the central cation in further QM/MM calculations, we have expanded the QM region by including now three ions closest to the H_2_ atom and two ions closest to both the H_4_ and H_5_ atoms, thus seven additional ions in total. Further reduction of the shielding constants of atoms H_2_, H_4_, and H_5_ is observed in Table S1. To calculate the ^1^H NMR spectrum of neat [C4mim][Cl] IL consistently, we have decided to perform a series of QM/MM calculations where the entire first solvation shell of the C4mim^+^ cation is treated quantum mechanically. To achieve this, we have included into the QM region all ions which have at least one atom with a distance not exceeding 4 Å from atoms H_2_, H_4_, or H_5_ or from carbon atoms at positions 6, 8, 9, or 10. The amount of cations and anions promoted to the QM region are in the ranges of 9–14 and 3–7, respectively, with averaged numbers being, respectively, 11.1 and 4.9. Within the 4 Å sphere centered specifically at atom H_2_, one or two anions were always found, and the average number is 1.46 anions. The latter value compares excellently with the coordination number of chloride anions around the atom H_2_ of 1.47; thus, the coordination of the C_2_–H_2_ moiety by the chloride anions as seen in the entire trajectory is reflected here in the reduced set of 100 configurations very well. The averaged number of anions coordinating both atoms H_4_ and H_5_ is 2.24, and in most cases, two or three anions are included into the QM region around this edge of the imidazolium ring.

As can be seen in Table S1, the results of these large-scale QM/MM calculations suggest that the shielding constants of hydrogen atoms H_2_, H_4_, and H_5_ obtained previously by the scheme where only the seven additional ions around the imidazolium ring were promoted to the QM region are virtually converged with respect to the expansion of the QM region. Quantum mechanical treatment of ions around the methyl and butyl groups has small yet notable and qualitatively different effect on the shielding constants of hydrogen atoms in these moieties. Compared to the case where the entire environment is treated classically, the shielding constants of hydrogens at positions 6, 7, and 8 are reduced by 0.36, 0.25, and 0.10 ppm, respectively, while those for hydrogens at positions 9 and 10 are increased by 0.09 and 0.06 ppm, respectively. Finally, we have also tested the effect of ions beyond the first solvation shell on the shielding constants of the central cation. We have achieved this by repeating the large-scale calculations where the first solvation shell is described quantum mechanically again, but all other ions are removed completely. Comparing the results of these two models as given in Table S1, we are in a position to confirm that the ^1^H NMR spectrum of neat [C4mim][Cl] IL is indeed essentially determined by the ions in the first solvation shell of the imidazolium cation, as was assumed previously.^39,44^

**Figure 5:**
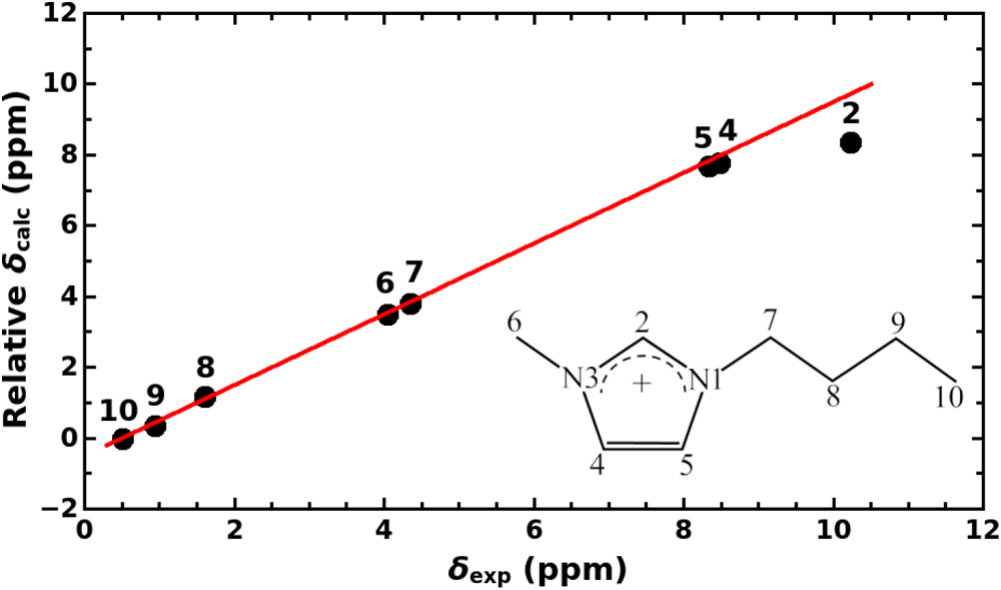
Calculated relative versus experimental ^1^H NMR spectrum of neat [C4mim][Cl] IL at 298 K. The unit line is drawn to facilitate the comparison between calculated and measured chemical shifts: points above the unit line indicate the overestimated chemical shift and vice versa. Atom numbering in the C4mim^+^ cation is included as an inset.

In Figure 5, we show the comparison between the experimental and the predicted relative ^1^H NMR spectra of neat [C4mim][Cl] IL.

The calculated spectrum is based on the shielding constants obtained by our most extensive QM/MM calculations, and relative chemical shifts were evaluated with respect to the shielding constant of hydrogen atoms at position 10 in the imidazolium cation.^74^ The experimental ^1^H NMR spectrum was measured for the sample of [C4mim][Cl] with a molar fraction of the IL of 0.98 in this work, and it is shown in Figure S7 of the Supporting Information. As can be seen in Table S1, the ^1^H NMR chemical shifts measured in this work agree with the corresponding values for neat [C4mim][Cl] IL reported in ref (33) to a few hundredth of the ppm. As can be seen in Figure 5, the agreement between the calculated and experimental spectra of neat IL is very good in both qualitative and quantitative terms. Even the tight spacing of 0.1 ppm between the signals of atoms H_4_ and H_5_ is reproduced correctly. Interestingly, this would not be the case if the effect of the ions beyond the first solvation shell were neglected, as can be seen in Table S1 of the Supporting Information. However, the model is found to underestimate the chemical shift of the H_2_ atom by a substantial 1.4 ppm. Considering the high quantitative accuracy for all other NMR signals delivered by the present QM/MM model and keeping in mind the high sensitivity of the H_2_ chemical shift on the location of the H_2_-atom-coordinating chloride anion with respect to the C_2_–H_2_ bond, we are inclined to argue that this discrepancy is caused by the incorrect angular distribution of the chloride anions around the C_2_–H_2_ moiety as shown in Figure 3a. Apparently, this distribution should be shifted more toward higher values of the angle and thus to more linear C_2_–H_2_··· Cl^–^ hydrogen bonds—an issue which was also discussed previously.^43^ Our results thus suggest that a refinement of the force field used in the present MD simulations of neat IL may be necessary in order to improve the local distribution of ions around the C_2_–H_2_ moiety of imidazolium cations.

### Mixtures of [C4mim][Cl] and Water

#### Structural Analysis

Turning to the mixtures of [C4mim][Cl] and water, the RDFs shown in Figures 2a as well as in S1 and S2 of the Supporting Information suggest that a small water admixture of χ_w_ = 0.05 has negligible effect on the local distribution of anions around the imidazolium ring. At this low content of water in the mixture, isolated water molecules are scattered across the simulation box, and they are found to form hydrogen bonding interactions with chloride anions exclusively, as signified by two pronounced peaks in the range of 1.7 to 4.0 Å in the RDF between Cl^–^ and hydrogen atoms of water molecules shown in Figure 2b. We thus find water molecules to act as hydrogen-bonded links between chloride anions as also seen in previous MD simulations of mixtures between other imidazolium ILs and water.^19,20,22^

**Figure 6:**
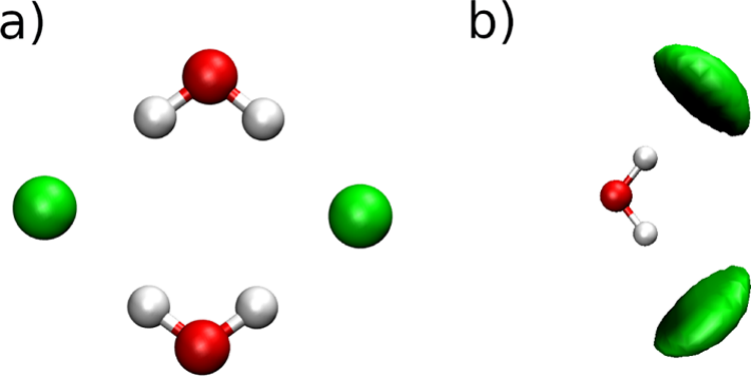
(a) Structure of a typical aggregate between Cl^–^ anions and water molecules and (b) SDF of Cl^–^ anions around the water molecule in the mixtures with χ_IL_ of 0.50 and 0.25.

The shape of the RDF illustrated in Figure 2b remains virtually the same when the molar fraction of the IL drops to 0.50. Spherical integration of the first peak in these RDFs indicates that O–H bonds in water molecules form on an average 1.00 and 0.98 hydrogen bonds with chloride anions when the molar fraction of the IL is equal to 0.95 and 0.50, respectively. Visual inspection of the recorded trajectory for the mixture with χ_IL_ = 0.50 reveals a very characteristic structural pattern for water-anionic aggregates where typically two water molecules share two chloride anions simultaneously in the planar arrangement as illustrated in Figure 6a and corroborated by the SDF of Cl^–^ anions around the water molecule shown in Figure 6b.

Furthermore, these aggregates are also seen to conglomerate into longer polymer-like chains which are situated in the areas in-between the cations. The interactions between anions and water molecules are in fact favored so much that the hydrogen bonding between water molecules is virtually absent as also suggested by the low intensity of the peak in the range of 1.6–2.3 Å in the RDF between the hydrogen and oxygen atoms of water molecules shown in Figure 2c for χ_IL_ = 0.50. The proliferation of water-anionic aggregates is accompanied by simultaneous exchange of anions by water molecules in the vicinity of the imidazolium ring as evident from the RDFs shown in Figures 2a, S1, and S2. This effect is also corroborated by the considerably increased intensity of the first peak in the RDF between H_2_ and oxygen atoms of water molecules shown in Figure 2d when χ_IL_ decreases from 0.95 to 0.50.

When the molar fraction of water rises to 0.75, extensive chains of water-anionic hydrogen-bonded aggregates of the type ··· Cl^–^··· (H–O–H)_*n*_··· Cl^–^··· with *n* equal to 1, 2, or 3 are observed, which are seen to surround aggregates of C4mim^+^ cations, thus leading to a strongly heterogeneous structure of the mixture.^13^ Indeed, the RDFs between C10 atoms of imidazolium cations shown in Figure S10 reveal that aggregation of the butyl moieties of C4mim^+^ is stimulated by the rising content of water in the mixture. Hydrogen bonding between water molecules is now also observed evidently, as the coordination number of Cl^–^ anions around the O–H bonds in water molecules drops to 0.75 at χ_IL_ = 0.25. Furthermore, the peak around 1.8 Å in the RDF between hydrogen and oxygen atoms of water molecules shown in Figure 2c is seen to gain substantial intensity when the molar fraction of water increases to 0.75. However, no indication of the formation of the water pockets—that is, isolated long-lived accumulations of water molecules—is found.

**Figure 7:**
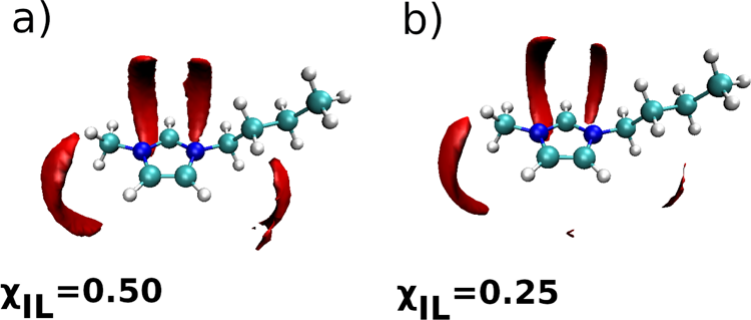
SDF of oxygen atoms of water molecules around the imidazolium ring of the C4mim^+^ cation in the mixtures with χ_IL_ of (a) 0.50 and (b) 0.25.

As evident from the RDFs shown in Figures 2a,d as well as S1, S2, S8, and S9, the exchange between chloride anions and water molecules around the imidazolium ring is intensified by the rising molar fraction of water. The coordination number of chloride anions around the C_2_–H_2_ moiety drops from 1.47 in neat IL to 1.37 and 1.14 in mixtures with χ_IL_ of 0.50 and 0.25, respectively. The RDFs between H_2_ atoms of the imidazolium cations and oxygen atoms of water molecules in the simulated mixtures shown in Figure 2d support this finding, and spherical integration of the first peak in these RDFs up to 4 Å give the coordination number of 1.21 and 2.73 in systems with χ_IL_ values of 0.50 and 0.25, respectively. Noteworthily, water molecules are found to approach the C_2_–H_2_ moiety of C4mim^+^ cations already in the mixture with χ_IL_ = 0.95, where the coordination number of oxygen atoms of water molecules by the H_2_ atoms of C4mim^+^ cations was integrated to be as high as 1.74. Water molecules are thus indeed seen to effectively screen electrostatic interactions between cations and anions in the mixture. Indeed, the SDF of oxygen atoms of water molecules around the C4mim^+^ cation shown in Figure 7 indicates that water molecules tend to replace the anions in the vicinity of the imidazolium ring.

Interestingly, water molecules and chloride anions show preference to virtually occupy the same areas around the imidazolium ring, compare the SDFs shown in Figures 4 and 7.

Water is also found to affect the structural distribution of anions around the C–H bonds in the imidazolium ring. As illustrated in Figure 3a, a clear trend for the distribution of angles between C_2_–H_2_ bond and chloride anions to shift to higher values is observed with increasing content of water. Simultaneously, chloride anions show an increased trend to also be located in the plane of the imidazolium ring in these mixtures as the pronounced peaks around ±120 deg in the distribution of the N_1_–C_2_–H_2_··· Cl^–^ dihedral angle shown in Figure 3b decrease, and the distribution rises in the areas around 0 and 180 deg instead. These structural changes are clearly visible in the SDF of Cl^–^ around the imidazolium ring calculated for the IL/water mixtures as shown in Figure 4, parts b and c. Structural changes around the C_4_–H_4_ and C_5_–H_5_ moieties induced by the rising content of water are briefly discussed in the Supporting Information.

#### NMR Results

The heterogeneous nature of the three-component mixture between [C4mim][Cl] IL and water as well as the slow dynamics of the ions and water molecules at ambient conditions implies inherently complex chemical equilibrium in these systems. Therefore, computations of the ensemble averages of the shielding constants which would properly reflect the rich phase space of these systems allowing for plausible comparison between computational results and experimental data are extremely difficult. In this work, we will resort to calculations of NMR shieldings for specific types of water-ionic aggregates, and our computational results are expected to be of sufficient quality in order to provide well-motivated rationalizations of the experimental data shown in Figure 1.

##### NMR Shielding Constants of Water

In Table 1, we present computational QM/MM results for the ^1^H NMR shielding constants of water molecules in the aqueous mixtures of [C4mim][Cl] IL. Distinct series of QM/MM calculations have been assigned IDs which are listed in the second column in Table 1. We refer to Figure S11 of the Supporting Information for a visualization of quantum mechanically treated subsystems in series **A** to **F**.

**Table 1:** Calculated ^1^H NMR Isotropic Shielding Constants of Water Molecules, σ in ppm, in Liquid Water and Its Mixtures with [C4mim][Cl] IL as Averages over 100 configurations^a^

χ_w_	ID	species around H_2_O	σ_a_	σ_b_
0.05	**A**	*H*_a_: 1 Cl^–^; *H*_b_: 1 Cl^–^; O: none	26.44 (0.10)	26.84 (0.11)
		*H*_a_: 1 Cl^–^; *H*_b_: 1 Cl^–^; O: 2 C4mim^+^	25.79 (0.11)	26.15 (0.11)
	**B**	*H*_a_: 1 Cl^–^; *H*_b_: 1 Cl^–^; O: 2 C4mim^+^	27.17 (0.12)	26.66 (0.11)
0.50	**C**	*H*_a_: 1 H_2_O; *H*_b_: 1 Cl^–^; O: 2 C4mim^+^	27.82 (0.11)	27.21 (0.11)
0.75	**D**	*H*_a_: 1 H_2_O; *H*_b_: 1 H_2_O; O: 2 C4mim^+^	28.67 (0.12)	28.54 (0.10)
	**E**	*H*_a_: 1 H_2_O; *H*_b_: 1 H_2_O; O: 1 H_2_O, 1 C4mim^+^	28.00 (0.10)	28.84 (0.12)
1.00	**F**	*H*_a_: 3 H_2_O; *H*_b_: 3 H_2_O; O: 4 H_2_O	28.20 (0.09)	

a Statistical errors are evaluated as standard deviations of the sample and are provided in parentheses. In the case of the IL/water mixtures, shielding constants have been calculated for each proton of a water molecule separately, designating the individual protons and the corresponding shielding constants as *H*_a_ and *H*_b_, and σ_a_ and σ_b_, respectively. Column 3 contains information concerning the nature and amount of different species included into the QM region, which are closest to each atom of the central water molecule.

The evolution of the ^1^H NMR chemical shift of water with the changing composition of the mixture is here referenced against the shielding constant of pure water. The ^1^H NMR shielding constant of pure water was calculated as an average over 100 molecular snapshots, and 10 additional water molecules closest to the randomly selected central water molecule were included into the QM region in each snapshot. The present approach to obtain the value for the ^1^H NMR shielding constant of pure water follows closely the computational scheme implemented in ref (58) where very accurate predictions of both ^1^H and ^17^O NMR shielding constants of pure water were obtained. The calculated value of the ^1^H NMR shielding constant of pure water designated as system **F** is included in Table 1.

First, we have performed a series of test calculations which have led to a clear conclusion that the previously used cost-effective strategy to utilize a small 3-21G basis set for the quantum mechanically described environment of the C4mim^+^ cation is not valid for the computations of the shielding constants of water molecules in the IL matrix, and the electronic structure of the entire QM region has to be described on an equal footing by using the same def2-TZVP basis set. In line with the high viscosity of the [C4mim][Cl] IL, we have observed that tumbling of the water molecules is hindered completely in the simulated mixtures, at least on the time scale of the production run that is 5 ns in this work. Therefore, we have provided statistical averages of the ^1^H NMR shielding constants for each hydrogen atom of the water molecule separately in Table 1.

In the case of the mixture with χ_IL_ = 0.95, we have performed two series of QM/MM calculations on a randomly selected water molecule, system **A** in Table 1. In the first series, the two chloride anions forming hydrogen bonding to both the O–H bonds of the central water molecule were considered at the QM level, while in the second series, the QM region was further expanded to also include two C4mim^+^ cations nearest to the oxygen atom site of the water molecule as well. As can be seen in Table 1, the effect of the quantum mechanical treatment of the ions in the immediate vicinity of the water molecule is mandatory to obtain correct values of the ^1^H NMR shieldings of water in the IL. In all subsequent QM/MM calculations on water molecules in the IL–water mixtures, we have subsequently included one species closest to each of the protons of the water molecule as well as two species closest to its oxygen atom into the QM region of the model. In the system with the lowest admixture of water, we have also performed QM/MM calculations on another randomly selected water molecule using the same computational scheme, refer to system **B** in Table 1. As evident from Table 1, the time averages of the shielding constants for each proton in the same water molecule differ by as much as 0.5–0.7 ppm, and this is a consequence of hindered rotation of water molecules—which in turn is apparently caused by the strong hydrogen bonding interactions between water molecules and Cl^–^ anions—as well as of local strongly anisotropic environment of the two O–H bonds of the water molecules. We also find that the ^1^H NMR shieldings of the two water molecules **A** and **B** are also rather different. Again, this is caused by the difference in the local environment of the two water molecules as in the case of system **A**, the two quantum mechanically treated cations are found to form hydrogen bonds with the water molecule through their C_2_–H_2_ moieties, whereas in system **B**, the C_5_–H_5_ bond of the first and the butyl group of the second imidazolium cation are found to coordinate the water molecule.

Compared to the shielding constant computed for pure water, system **F**, both water molecules in the mixture with χ_IL_ = 0.95 display substantially lower values for their ^1^H NMR shielding constants, thereby implying larger chemical shifts for water molecules in the mixture than in pure water. This allows concluding that the rising curve of the chemical shift of water observed in Figure 1a when molar fraction of water streams to zero is due to the rising relative population of water molecules which form hydrogen bonding with the chloride anions. As discussed above, the water molecules still form prolific aggregates with the chloride anions in the equimolar mixture of [C4mim][Cl] IL and water, and hydrogen bonding between water molecules is rather scarce in this system. We have thus performed QM/MM calculations for the water molecule whose O–H bonds form hydrogen bonding with another water molecule and with a chloride anion, that is system **C**. As evident from Table 1, the shielding constant computed for the hydrogen atom of the water molecule which is involved in hydrogen bonding with another water molecule is larger than for that which is involved in the hydrogen bonding with the anion. However, that shielding constant computed for the hydrogen atom involved in hydrogen bonding with another water molecule is still smaller than the corresponding value calculated for pure water. This implies a larger ^1^H NMR chemical shift of the water molecule in the mixture than in pure water, thus in contradiction to the experimental data seen in Figure 1a.

Turning to the mixture with largest content of water, χ_w_ = 0.75, we have performed two series of QM/MM calculations on water molecules referred to as systems **D** and **E**, yet both water molecules form hydrogen bonds to other water molecules. In system **E**, the central water molecule also acts as a hydrogen bond acceptor for the third water molecule, while in system **D**, two C4mim^+^ cations are found to be closest to the oxygen atom of the central water molecule. Noteworthily, all water molecules found in the vicinity of the central water molecule form in turn hydrogen bonding with chloride anions. As can be seen in Table 1, the computed ^1^H NMR shielding constants of water molecules are substantially larger than that of pure water in three cases out of four. We thus have found important computational evidence that the lowering of the chemical shift of water in aqueous mixtures of [C4mim][Cl] as compared to that of pure water as can be seen in Figure 1a has to be caused by the water molecules which are free from hydrogen bonding with chloride anions and possibly are found in the second solvation shell of the chloride anions. We would like to note that the minimum chemical shift of water was recorded for the mixture with χ_IL_ = 0.34, see Figure 1a. However, water-anionic aggregates are seen to strongly prevail over the hydrogen-bonded aggregates of water molecules even in the simulated mixture with χ_IL_ = 0.25. We thus have to conclude that the force fields selected for the IL and water in the present work are favoring water-anionic interactions too much, thus adding to the previously raised concerns that the present force field applied for the IL as well as for water molecules may not be able to describe the intermolecular structure of the studied IL/water mixtures properly.

##### NMR Shielding Constant of the H_2_ Atom of the C4mim^+^ Cation

In Table 2, we have collected the QM/MM results for the ^1^H NMR shielding constants of the H_2_ atom of the C4mim^+^ cation computed for the neat [C4mim][Cl] IL and for its mixtures with water. We refer to Figure S12 of the Supporting Information for a visualization of quantum mechanically treated subsystems in series **G** to **M**.

**Table 2:** Calculated ^1^H NMR Isotropic Shielding Constants of the H_2_ Atom of C4mim^+^ Cations, σ in ppm, in Neat [C4mim][Cl] IL and Its Mixtures with Water as Averages over 100 configurations^a^

		species around the H_2_ atom				
χ_IL_	ID	species	Min #	Max #	Aver. #	σ
1.00	**G**	Cl^–^	1	2	1.46	22.53 (0.09)
		C4mim^+^	2	6	3.19	
0.50	**H**	Cl^–^	1	2	1.20	22.69 (0.05)
		C4mim^+^	0	3	0.83	
		H_2_O	3	6	4.43	
	**I**	Cl^–^	0	3	2.04	22.92 (0.05)
		C4mim^+^	0	3	1.39	
		H_2_O	2	5	3.94	
	**J**	Cl^–^	0	2	0.23	23.77 (0.08)
		C4mim^+^	1	5	2.51	
		H_2_O	1	3	1.39	
0.25	**K**	Cl^–^	0	1	0.99	23.00 (0.05)
		C4mim^+^	0	4	1.47	
		H_2_O	2	6	4.57	
	**L**	Cl^–^	1	2	1.84	23.14 (0.06)
		C4mim^+^	0	4	1.33	
		H_2_O	2	7	4.65	
	**M**	Cl^–^	0	1	0.01	23.80 (0.08)
		C4mim^+^	0	3	1.45	
		H_2_O	1	3	1.84	

a Statistical errors are evaluated as standard deviations of the sample and are provided in parentheses. Columns 3–6 give statistical information concerning the nature and amount of different species found around atom H_2_ of the central cation within the distance of 4 Å.

As evident from Table 2, the value of the computed ^1^H NMR shielding constant of the H_2_ atom in the imidazolium cation varies in the range of about 1 ppm depending on the structure of the local environment around the C_2_–H_2_ bond. However, coordination by chloride anions seems to be the major factor determining its value. For mixtures with χ_IL_ values of 0.50 and 0.25, the shielding constants computed for systems **H**, **I**, **K**, and **L** with averaged number of chloride anions in the range of about 1–2 lead to rather similar values for the shielding constant. The computed shielding constants of the H_2_ atom are still larger in the mixtures than in the neat IL, even in those cases where the averaged number of chlorides around the C_2_–H_2_ bond is larger than in the neat [C4mim][Cl]. These results are in line with the observed decrease of the chemical shift of atom H_2_ with the increasing molar fraction of water as can be seen in Figure 1b. In systems **J** and **M** with low averaged number of chloride anions near the C_2_–H_2_ moiety, a substantial increase in the shielding constant is predicted by as much as 1.3 ppm as compared to the value computed for the neat IL, system **G**. The present results are in line with the previous observations that the presence or the absence of the hydrogen bonding between the C_2_–H_2_ group and the chloride anion is the main factor determining the H_2_ shielding constant of the imidazolium cation in the liquid phase.^49^ Our computational results in Table 2 thus allow concluding that the observed monotonically decreasing chemical shift of the H_2_ atom with the increasing content of water in the mixture as illustrated in Figure 1b is due to the gradual breakdown of the hydrogen bonding between the C_2_–H_2_ moiety of imidazolium cations and chloride anions. Notably, imidazolium chloride ILs are known to eventually dissociate into free fully solvated ions at the conditions of infinite dilution in an aqueous solution.^49^

## Summary

A peculiar non-monotonic dependence of the ^1^H NMR chemical shift of water on the composition of the aqueous mixtures of the [C4mim][Cl] IL has been recorded in this work. When the molar fraction of the IL rises from 10^–3^ to 0.34, the chemical shift of water is seen to decrease from 4.8 to 4.2 ppm. Then, this chemical shift starts to increase reaching a value of 4.5 ppm at 0.98 molar fraction of the IL. The ^1^H NMR chemical shift of the H_2_ atom in the C4mim^+^ cation exhibits rather high sensitivity to the composition of the mixture as well, rising gradually from 8.7 to 10.2 ppm when the molar fraction of the IL is varied from 10^–3^ to 0.98.

These experimental findings reflect the shifting equilibrium between various ionic, water-ionic, and water–water aggregates with the changing composition of the mixture. To understand the structural organization of the [C4mim][Cl]/water mixtures, we invoke here an integrated theoretical approach which combines classical MD simulations and QM/MM calculations of NMR shielding constants. Extensive MD simulations of the neat [C4mim][Cl] IL and of its mixtures with water have been conducted, and structural analysis of the recorded trajectories was performed. The ^1^H NMR shielding constants of the water molecules and of the C4mim^+^ cations were computed using the QM/MM scheme for sets of molecular configurations extracted from the trajectory, and the liquid-state results for shielding constants were obtained as statistical averages over the configurations.

The computed relative ^1^H NMR spectrum of neat [C4mim][Cl] IL is found to be in a very good qualitative and quantitative agreement with the experimental data. The largest discrepancy was observed for the chemical shift of the H_2_ atom of C4mim^+^, which has been underestimated by 1.4 ppm. This discrepancy can be attributed to the likely inaccurate local distribution of Cl^–^ anions around the C_2_–H_2_ moiety of the C4mim^+^ cations in the neat IL that may be induced by the imperfections of the force field used in our MD simulations. We have found computational evidence that the decreased chemical shift of water molecules in the [C4mim][Cl]/water mixtures as compared to the chemical shift of neat water is due to the water molecules which form hydrogen bonding to other water molecules and not to the hydrophilic Cl^–^ anions. In contrast, the QM/MM calculations predict larger chemical shift of water molecules involved in the aggregates with the anions as compared to that of neat water. Because the relative population of the hydrogen-bonded aggregates between chloride anions and water molecules grows with the diminishing molar fraction of water, the overall chemical shift of water should increase, as indeed observed experimentally when the molar fraction of the IL rises from 0.34 to 0.98. The computations also have demonstrated that the decreasing chemical shift of the H_2_ atom in the C4mim^+^ cations observed with the rising molar fraction of water in the mixture is due to the gradual replacement of chloride anions by water molecules in the vicinity of the C_2_–H_2_ moiety.

## Supporting Information Available

Additional structural analysis in relation to the distribution of ions and water molecules around the C_4_–H_4_ and C_5_–H_5_ moieties of C4mim^+^ cations; H_4_–Cl^–^ and H_5_–Cl^–^ RDFs recorded for the neat [C4mim][Cl] IL and for its mixtures with water; angular distribution of Cl^–^ anions around the C_4_–H_4_ and C_5_–H_5_ moieties of C4mim^+^ cations recorded for the neat [C4mim][Cl] IL and for its mixtures with water; experimental ^1^H NMR spectrum of the [C4mim][Cl] IL at 298 K; H_4_–O_w_ and H_5_–O_w_ RDFs recorded for the aqueous mixtures of [C4mim][Cl] IL; C_10_–C_10_ RDFs recorded for the neat [C4mim][Cl] IL and for its mixtures with water; visualization of the QM region in the QM/MM calculations for systems of neat [C4mim][Cl] IL and of its mixtures with water; and computational and experimental ^1^H NMR results for neat [C4mim][Cl] IL (PDF)
