# Supplementary material for: Structural Features of the [C4mim][Cl] Ionic Liquid and Its Mixtures with Water: Insight from a 1H NMR Experimental and QM/MD Study
Source: J Phys Chem B. 2021 Nov 22;125(48):13255–66. doi: 10.1021/acs.jpcb.1c08215 (PMC8667039; doi:10.1021/acs.jpcb.1c08215)
Supplement: Supplementary file 1 — jp1c08215_si_001.pdf [file jp1c08215_si_001.pdf]

Dated: November 9, 2021

Supporting Information for:

Structural Features of the [C4mim][Cl] Ionic Liquid and Its Mixtures with Water: Insight from a ^{1}H NMR Experimental and QM/MD Study

by

Dovilė Lengvinaitė,[†] Sonata Kvedaravičiūtė,[‡] Stasė Bielskutė,[†,@] Vytautas Klimavicius,[†] Vytautas Balevicius,[†] Francesca Mocci,[¶] Aatto Laaksonen,[§,‖,⊥,#] and Kęstutis Aidas[†,*]

† Institute of Chemical Physics, Faculty of Physics, Vilnius University, LT-10257 Vilnius, Lithuania

‡ DTU Chemistry, Technical University of Denmark, DK-2800 Kongens Lyngby, Denmark

¶ Università di Cagliari, Dipartimento di Scienze Chimiche e Geologiche, Cittadella Universitaria di Monserrato, Monserrato, I-09042 Cagliari, Italy

§ Energy Engineering, Division of Energy Science, Luleå University of Technology, 97181 Luleå, Sweden

‖ Division of Physical Chemistry, Department of Materials and Environmental Chemistry, Arrhenius Laboratory, Stockholm University, Stockholm 10691, Sweden

⊥ Center of Advanced Research in Bionanoconjugates and Biopolymers, „Petru Poni" Institute of Macromolecular Chemistry, Iasi 700469, Romania

State Key Laboratory of Materials-Oriented and Chemical Engineering, Nanjing Tech University, Nanjing 211816, China

@ Present address: Institute for Research in Biomedicine (IRB Barcelona), The Barcelona Institute of Science and Technology, 08028 Barcelona, Spain

*E-mail: kestutis.aidas@ff.vu.lt, Phone: +370 5 223 4593.

1 Structural analysis

The H4-Cl$^-$ and H5-Cl$^-$ RDFs shown in Figures S1 and S2, respectively, exhibit similar structure as that of H2-Cl$^-$ RDF in Figure 2a of the article. The most probable H4/5$\cdots$Cl$^-$ hydrogen bond lengths are also equal to 2.67 Å, and spherical integration of H4-Cl$^-$ and H5-Cl$^-$ RDFs up to 4.0 Å gives the coordination numbers of 1.28 and 1.22, respectively, thus somewhat lower than the coordination number of chloride anions around the C2-H2 moiety.

The distribution of the C4-H4$\cdots$Cl$^-$ and C5-H5$\cdots$Cl$^-$ angles in neat [C4mim][Cl] IL shown in Figures S3 and S4, respectively, exhibit similar shapes as that seen for the distribution of the C2-H2$\cdots$Cl$^-$ angle shown in Figure 3a of the article. However, the most probable C4-H4$\cdots$Cl$^-$ and C5-H5$\cdots$Cl$^-$ angles are found to be around 140 deg in both cases, thus somewhat larger as compared to the most probable angle of the C2-H2$\cdots$Cl$^-$ hydrogen bond. Part of the anions are found to be situated on top of the C4-H4 and C5-H5 bonds, and linear hydrogen bonds are rare. The distribution of the C5-C4-H4$\cdots$Cl$^-$ and C4-C5-H5$\cdots$Cl$^-$ dihedral angles illustrated in Figures S5 and S6, respectively, show pronounced maxima at ±120 deg just as seen for the distribution of the N1-C2-H2$\cdots$Cl$^-$ dihedral angle in Figure 3b of the article, thus the preference for the chlorides to be located either on the butyl or the methyl side with equal probabilities. In addition, the probability for the anions to be located in the plane of the imidazolium ring are higher in these cases as compared to the corresponding case where anions are found in the vicinity of the C2-H2 bond. The distributions seen in Figures S5 and S6 are rather different in the range of -90 to +90 deg as compared to that shown in Figure 3b. They suggest that chloride anions are also often found in the area in-between C4-H4 and C5-H5 bonds, and they prefer to be located roughly in the plane of the imidazolium ring in this case.

Unlike for the case of distribution of chloride around the C2-H2 moiety, the angular distributions of chloride anions around the C4-H4 and C5-H5 bonds shown in Figures S3 and S4, respectively, are less sensitive to the amount of water in the mixture. However, the dihedral angle distributions shown in Figures S5 and S6 indicate the increasing preference for the chloride anions to stay in the region in-between the C4-H4 and C5-H5 moieties with the rising content of water in the mixture. The populations of anions on the methyl and butyl sides are thus seen to diminish, especially in the system with $\chi_{\rm IL} = 0.25$.

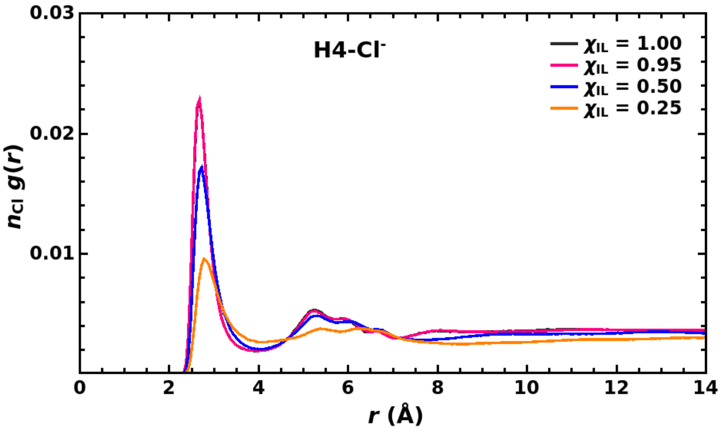

Figure S1: Radial distribution functions between H4 atom and Cl$^-$ ion in neat [C4mim][Cl] IL and its mixtures with water, scaled by the number density of the chloride anions, $n_{\rm Cl}$, for each specific system.

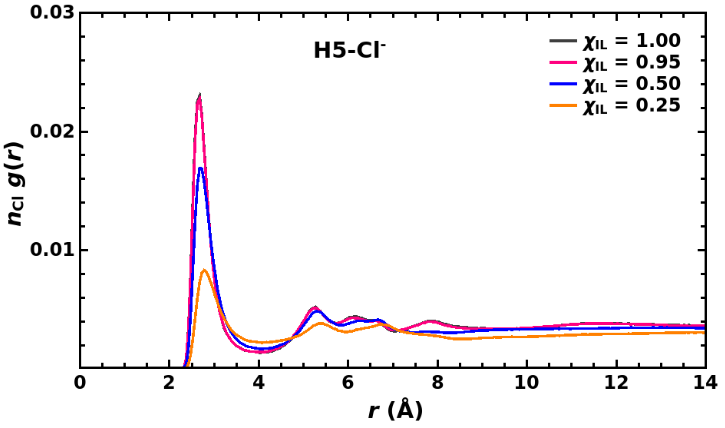

Figure S2: Radial distribution functions between H5 atom and Cl^- ion in neat [C4mim][Cl] IL and its mixtures with water, scaled by the number density of the chloride anions, n_{Cl}, for each specific system.

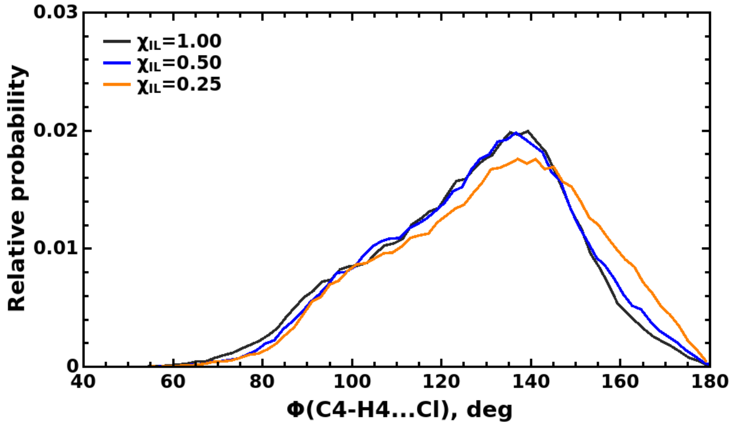

Figure S3: Distribution of the C4-H4$\cdots$$Cl^-$ angle, Φ, in neat [C4mim][Cl] IL and its mixtures with water for $R(\mathrm{H4}\cdots\mathrm{Cl}^-) \leq 4.0$ Å.

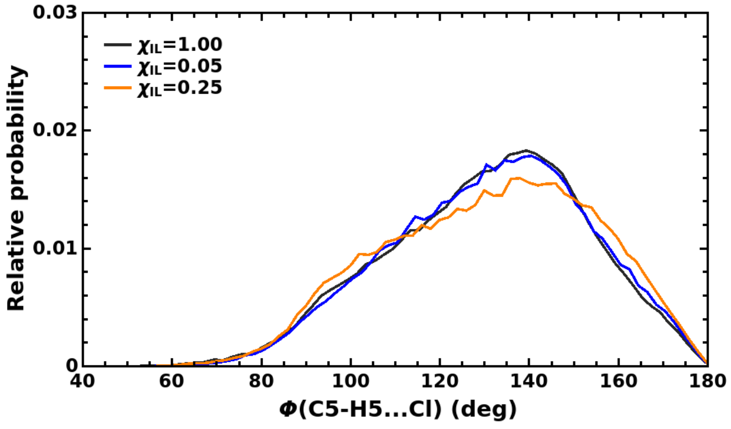

Figure S4: Distribution of the C5-H5$\cdots$$Cl^-$ angle, Φ, in neat [C4mim][Cl] IL and its mixtures with water for $R(\mathrm{H5}\cdots\mathrm{Cl}^-) \leq 4.0$ Å.

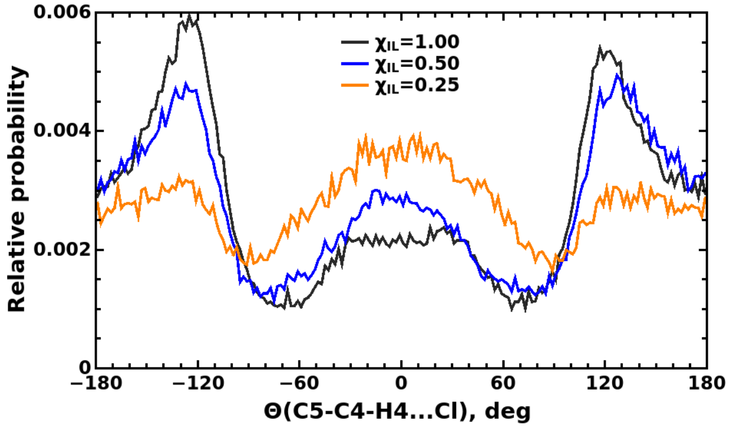

Figure S5: Distribution of the C5-C4-H4$\cdots$Cl$^-$ dihedral angle, Θ, in neat [C4mim][Cl] IL and its mixtures with water for R(H4$\cdots$Cl$^-$) $\leq$ 4.0 Å.

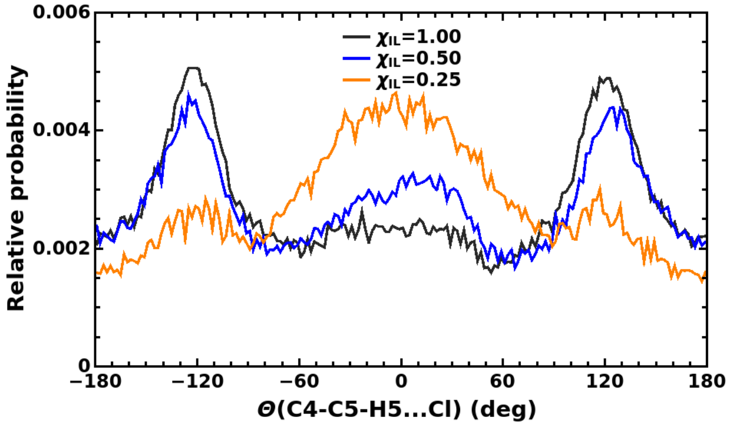

Figure S6: Distribution of the C4-C5-H5$\cdots$Cl$^-$ dihedral angle, Θ, in neat [C4mim][Cl] IL and its mixtures with water for R(H5$\cdots$Cl$^-$) $\leq$ 4.0 Å.

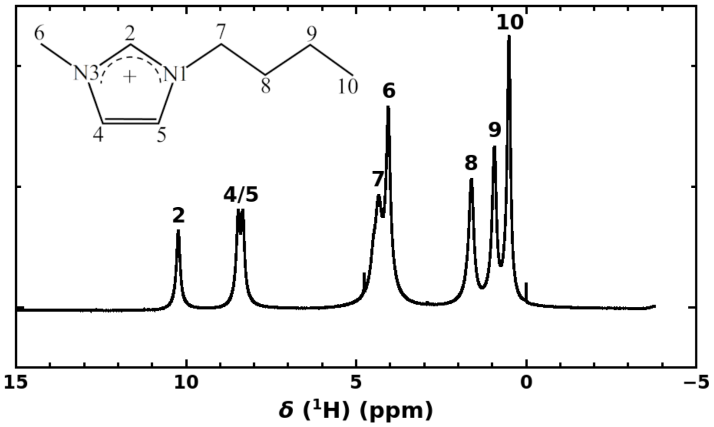

Figure S7: The ^{1}H NMR spectrum of a sample of [C4mim][Cl] with $\chi_{\rm IL}$ = 0.98 recorded at the temperature of 298 K. Atom numbering in C4mim$^+$ cation is included as an insert.

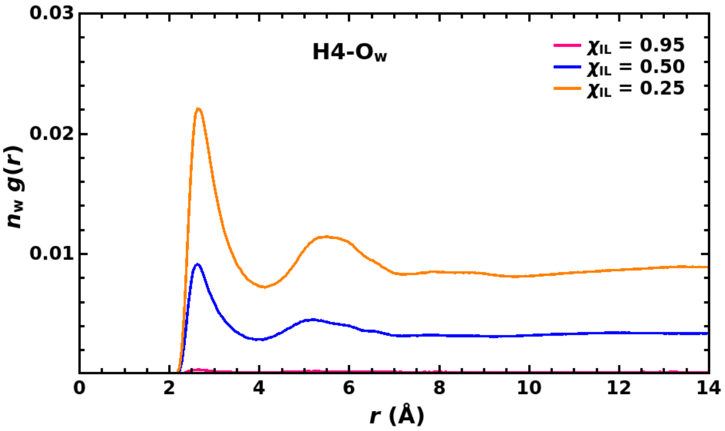

Figure S8: Radial distribution functions between H4 and oxygen atoms of water in mixtures of [C4mim][Cl] and water, scaled by the number density of the water molecules, n_w, for each specific system.

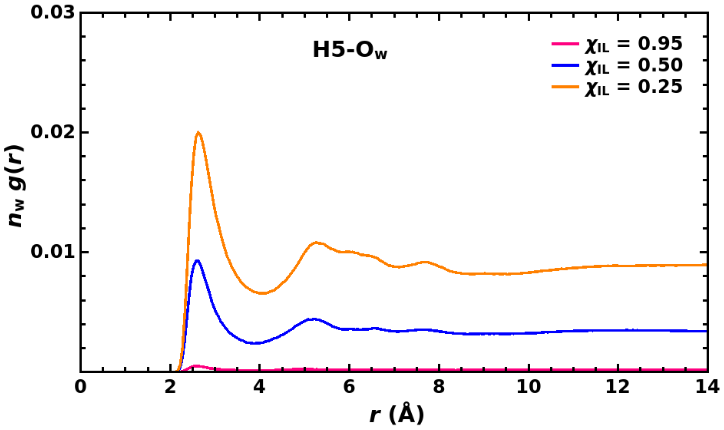

Figure S9: Radial distribution functions between H5 and oxygen atoms of water in mixtures of [C4mim][Cl] and water, scaled by the number density of the water molecules, n_w, for each specific system.

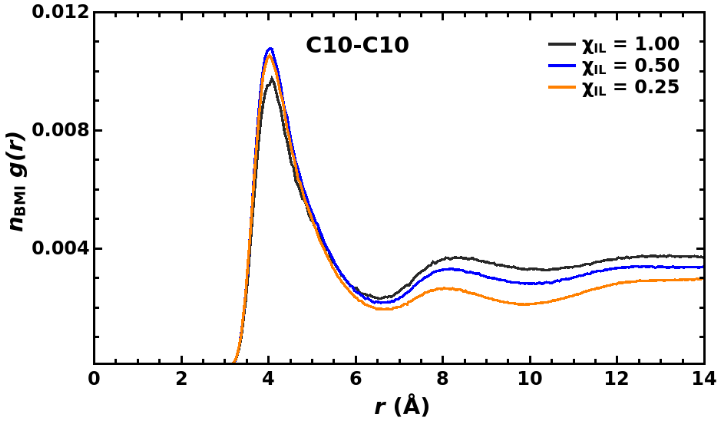

Figure S10: Radial distribution functions between C10 atoms of C4mim$^+$ cations in neat [C4mim][Cl] IL and its mixtures with water, scaled by the number density of the imidazolium cations, n_BMI, for each specific system.

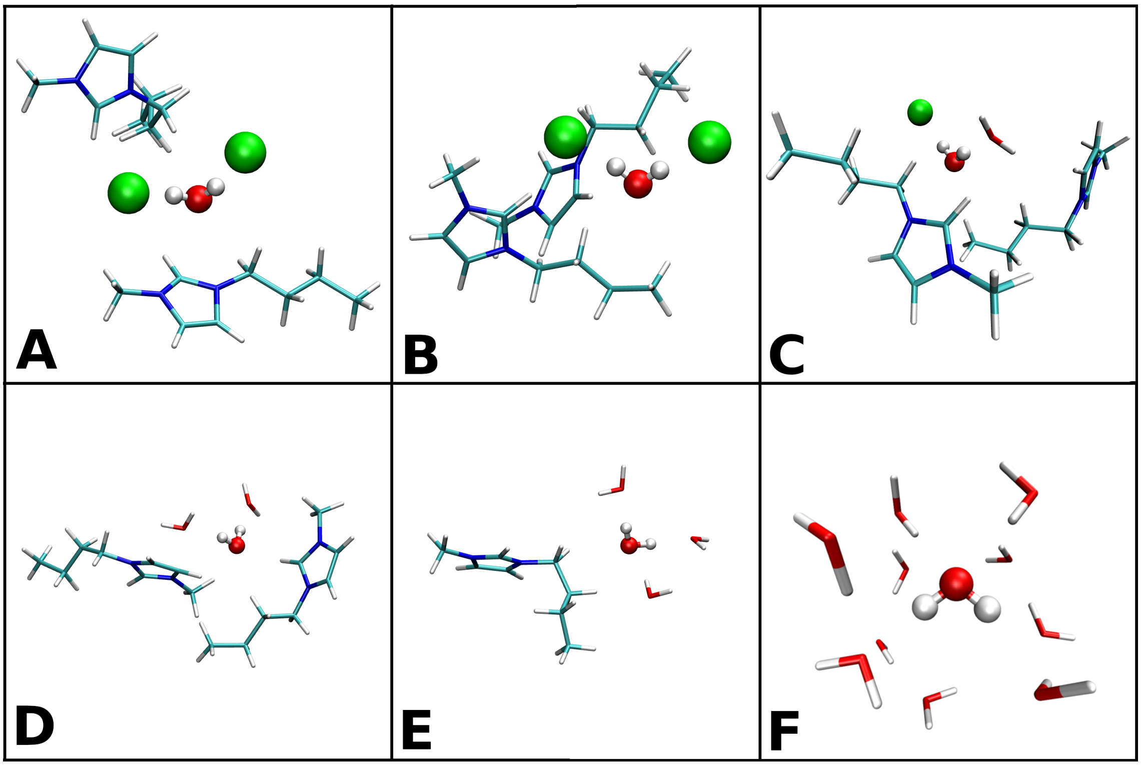

Figure S11: Visualization of a QM region in the QM/MM calculations for systems **A** to **F**, see main text of the article for details. Central water molecule is represented by the ball-and-stick model. Additional species included to the QM region around the central water molecule are represented by the stick model, except for the chloride anions which are represented as green balls. Ions and water molecules described by the point charges in the QM/MM calculations are not shown. Note that the geometry of each system is taken from a single randomly selected configuration; the structure of the quantum mechanically treated region changes from configuration to configuration in each specific system.

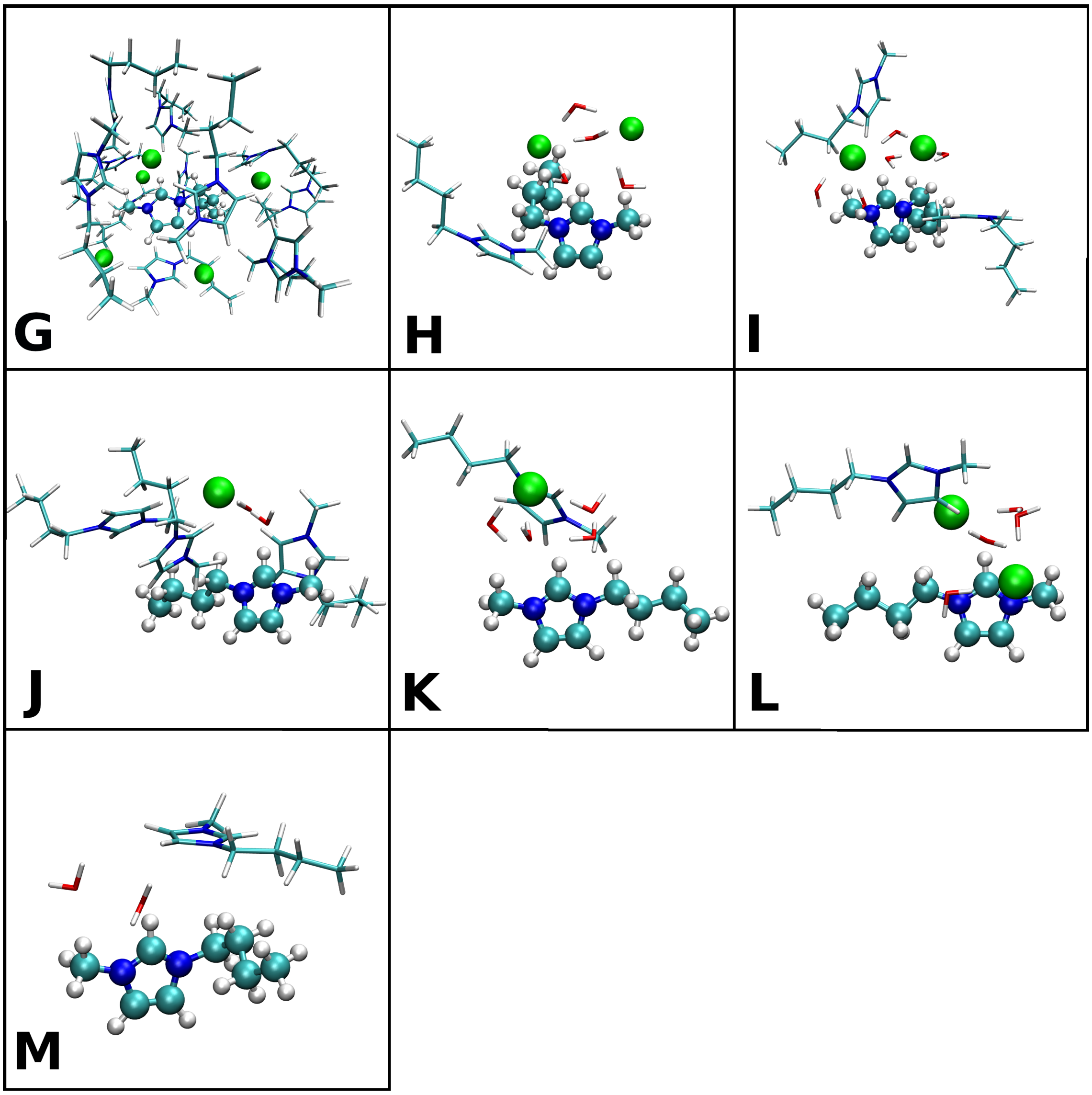

Figure S12: Visualization of a QM region in the QM/MM calculations for systems **G** to **M**, see main text of the article for details. Central C4mim$^+$ cation is represented by the ball-and-stick model. Additional species included to the QM region around the central cation are represented by the stick model, except for chloride anions which are represented as green balls. Ions and water molecules described by the point charges in the QM/MM calculations are not shown. Note that the geometry of each system is taken from a single randomly selected configuration; the structure and composition of the quantum mechanically treated region changes from configuration to configuration in each specific system. See Table 2 in the article for statistical information concerning the nature and amount of different species included to the QM region in these systems.

Table S1: The QM/MM based ^{1}H NMR isotropic shielding constants (in ppm) of $C4mim^+$ cation in pure [C4mim][Cl] IL simulated at the temperature of 298 K. The KT3 functional and def2-TZVP basis set were used in the QM/MM calculation unless stated otherwise. All entries for shielding constants are arithemtic averages over 100 molecular configurations with central cation selected randomly in each configuration. Statistical errors calculated as sample standard deviations are given in paranthesis. The first column indicates the number of ions included to the QM region along with the central cation. Here, the three integers given in parentheses indicate the numbers of ions promoted to the QM region closest to H2, H4, and H5 atoms, respectively. Second column indicates point-charge potential used for classical $C4mim^+$ cations. Refer to Figure 3 in the main text of the article for atom labeling in $C4mim^+$ cation. Experimental data for ^{1}H NMR chemical shifts of neat [C4mim][Cl] IL are listed in the last two lines of the table.

To QM	Pot.	H2	H4	H5	H6	H7	H8	H9	H10
(0,0,0)	—	24.01 (0.03)	24.34 (0.03)	24.30 (0.03)	27.81 (0.01)	27.49 (0.02)	29.79 (0.02)	30.45 (0.03)	30.62 (0.02)
(0,0,0)	RESP	23.59 (0.04)	23.98 (0.04)	23.99 (0.04)	27.77 (0.02)	27.35 (0.03)	29.83 (0.04)	30.46 (0.03)	30.84 (0.03)
(1,1,1)	RESP	23.08 (0.09)	23.57 (0.09)	23.57 (0.10)	27.73 (0.03)	27.32 (0.04)	29.81 (0.05)	30.44 (0.04)	30.84 (0.03)
$(1,1,1)^a$	RESP	23.04 (0.10)	23.53 (0.10)	23.54 (0.10)	27.74 (0.04)	27.33 (0.04)	29.82 (0.05)	30.45 (0.04)	30.86 (0.03)
$(1,1,1)^b$	RESP	23.19 (0.08)	23.70 (0.08)	23.70 (0.08)	27.77 (0.03)	27.37 (0.04)	29.85 (0.05)	30.48 (0.04)	30.86 (0.03)
$(3,2,2)^a$	RESP	22.44 (0.08)	23.22 (0.10)	23.23 (0.08)	27.63 (0.05)	27.20 (0.06)	29.79 (0.05)	30.45 (0.05)	30.93 (0.03)
$R \leq 4.0$ Å^{a,c}	RESP	22.53 (0.09)	23.21 (0.10)	23.11 (0.08)	27.41 (0.05)	27.10 (0.06)	29.73 (0.06)	30.54 (0.06)	30.90 (0.05)
$R \leq 4.0$ Å^{a,c}	—	22.61 (0.09)	23.02 (0.10)	23.08 (0.10)	27.39 (0.06)	27.14 (0.08)	29.84 (0.07)	30.60 (0.06)	31.03 (0.06)
Exp. δ (this work)^d		10.23	8.33	8.47	4.05	4.34	1.61	0.94	0.50
Exp. δ (Cha *et al.*)^e		10.22	8.34	8.47	4.08	4.36	1.63	0.96	0.53

^{a} – def2-TZVP basis for central cation, 3-21G basis for all additional ions.
^{b} – def2-TZVP basis for central cation, 3-21++G basis for all additional ions.
^{c} – all ions that are within the 4 Å distance from H2, H4 and H5 as well as from carbons 6, 8, 9 and 10 have been included to the QM region.
^{d} – ^{1}H NMR chemical shifts of [C4mim][Cl] ($\chi_{IL} = 0.98$) measured at 298 K in this work.
^{e} – ^{1}H NMR chemical shifts of neat [C4mim][Cl] measured at 298 K in Phys. Chem. Chem. Phys. 16 (2014), 9591. Numerical values were provided by Prof. D. Kim (Sogang University, Korea) through private communication.
